# The Molecular Mechanism of Ion-Dependent Gating in Secondary Transporters

**DOI:** 10.1371/journal.pcbi.1003296

**Published:** 2013-10-24

**Authors:** Chunfeng Zhao, Sergei Yu. Noskov

**Affiliations:** Center for Molecular Simulations, Department of Biological Sciences, University of Calgary, Calgary, Canada; UNC Charlotte, United States of America

## Abstract

LeuT-like fold Na-dependent secondary active transporters form a large family of integral membrane proteins that transport various substrates against their concentration gradient across lipid membranes, using the free energy stored in the downhill concentration gradient of sodium ions. These transporters play an active role in synaptic transmission, the delivery of key nutrients, and the maintenance of osmotic pressure inside the cell. It is generally believed that binding of an ion and/or a substrate drives the conformational dynamics of the transporter. However, the exact mechanism for converting ion binding into useful work has yet to be established. Using a multi-dimensional path sampling (string-method) followed by all-atom free energy simulations, we established the principal thermodynamic and kinetic components governing the ion-dependent conformational dynamics of a LeuT-like fold transporter, the sodium/benzyl-hydantoin symporter Mhp1, for an entire conformational cycle. We found that inward-facing and outward-facing states of Mhp1 display nearly the same free energies with an ion absent from the Na2 site conserved across the LeuT-like fold transporters. The barrier separating an apo-state from inward-facing or outward-facing states of the transporter is very low, suggesting stochastic gating in the absence of ion/substrate bound. In contrast, the binding of a Na2 ion shifts the free energy stabilizing the outward-facing state and promoting substrate binding. Our results indicate that ion binding to the Na2 site may also play a key role in the intracellular thin gate dynamics modulation by altering its interactions with the transmembrane helix 5 (TM5). The Potential of Mean Force (PMF) computations for a substrate entrance displays two energy minima that correspond to the locations of the main binding site S1 and proposed allosteric S2 binding site. However, it was found that substrate's binds to the site S1 ∼5 kcal/mol more favorable than that to the site S2 for all studied bound combinations of ions and a substrate.

## Author Summary

This study provides direct insights of how ion-dependent secondary transporters couples ion gradients to transport of a substrate. We conclude that the apo-form of a protein samples multiple conformational states separated by only small barriers. Binding of a single ion is sufficient to shift this conformational equilibrium towards one of the conformational states. Our work provides atomic detail for an ion-dependent gating that is at the heart of transport across membrane and present in many kingdoms of life.

## Introduction

Ion-coupled secondary-active transporters are integral membrane proteins involved in the cellular uptake and release of various substrates across cell membranes. These transporters transport the main substrate uphill against its concentration gradient by coupling with the favorable downhill transport of ion(s). Examples of their substrates include neurotransmitters (serotonin, dopamine, and epinephrine), sugars, amino acids, and nucleobases [1]–[3]. The malfunction of secondary transporters is implicated in various neurological, skin, cardiovascular, and renal diseases in humans [3]. As a result, ion-coupled secondary transporters are important targets for drugs in the treatment of psychotic states, schizophrenia, clinical depression, diabetes, and obesity [2], [3]. For example, several sodium-glucose co-transporter 2 (SGLT2) inhibitors are being developed as a new class of drugs to treat type 2 diabetes [4], [5], while transporters from the neurotransmitter:sodium symporter (NSS) family are common targets for anti-depressants (tricyclic antidepressants and selective serotonin reuptake inhibitors) and drug of abuse [6]–[9].

The generally accepted mechanism for secondary-active transport is the alternating access model [10] in which the transporter changes between outward-facing and inward-facing conformations, allowing the main substrate and coupled ion(s) to bind to one side and be released from the other side. The progress in structural studies of secondary transporters led to identification of several structure folds including a so-called “LeuT-like” fold. The LeuT-like fold secondary transporters share common structural features, including two-fold symmetric inverted repeats that involve 5+5 essential helices and a break in the TM helices that forms the substrate binding pocket(s) [1]–[3]. Recently, the sodium/hydantoin symporter Mhp1 [11], [12], the sodium/leucine symporter LeuT [13], [14], and the sodium/betaine symporter BetP [15], [16] were crystallized in both outward-facing and inward-facing conformational states, providing detailed molecular insight into the atomic structures of the key protein states involved in the transport of a substrate. Structurally, Mhp1 has the same “LeuT-like fold” as LeuT, vSGLT, BetP and several other transporters despite belonging to a different gene family [12] and thus represents an ideal target for understanding of basic principles that governs mechanism of secondary transport in LeuT-like fold transporters.

Mhp1 belongs to the nucleobase-cation-symport-1 gene family (NCS1). Similarly to LeuT, Mhp1 is Na^+^ dependent. Mhp1 has a transport stoichiometry of 1 Na^+^: 1 substrate, differing from the 2 Na^+^:1 substrate ratio in LeuT. Despite the different stoichiometry, the sodium-binding site in Mhp1, corresponding to the Na2 site in LeuT [14], is conserved in LeuT-like fold Na-dependent secondary transporters [3], [17], [18]. Thus, a detailed molecular-level understanding of the transport mechanism of Mhp1 may offer a chance to generalize the coupling mechanism for this large group of LeuT-like fold Na-dependent secondary transporters. The crystal structures for Mhp1 were captured in the following states: outward-facing open state with a bound sodium ion (*O_i*), outward-facing occluded state with a bound substrate and a sodium ion (*O_si*), and apo inward-facing open (*I_apo*) [11], [12]. Several tentative mechanisms were proposed recently by Shimamura *et al.* [11] with the use of a dynamical-importance sampling study for the transitions and Adelman *et al.* [19] extending a weighted ensemble path-sampling study with coarse-grained model. Although these studies offered a potential scheme for the conformational dynamics in Mhp1, the studies also have serious limitations. Most importantly, the transported Na^+^ and the benzyl hydantoin substrate, despite playing an essential role in the transport cycle [17], [20]–[24], were not included explicitly in the path sampling computations. Therefore one of the key questions in the mechanism of secondary transport remained untouched. In particular, how does the binding of a sodium ion stabilize different conformational states of the system? To aid the understanding of the role of the sodium ion in conformational transitions, we performed atomistic simulations for the whole transport cycle of *O_i*→*O_si*→*I_apo*→*O_i* with explicit membrane, water environment, and the transported ion and substrate. To elucidate a reaction path for this transition, we used the swarm-of-trajectories method of Pan *et al.* [25], [26]. The resulting minimum-energy reaction path was used then for detailed exploration of the energetics of the protein conformational transition as a function of ion/substrate binding with free energy simulations. Specifically, we report multiple 2-dimensional-potential of mean force (2D- PMF) profiles [27]–[29] along the gating reaction coordinate and discuss role of ion/ligand binding in stabilization of different conformational states of the system. These profiles help to elucidate the thermodynamic contributions governing the ion/ligand translocation and transporter conformational changes [30].

## Results/Discussions

### Defining the order parameters

One of the key challenges in characterizing the free energy landscapes of conformational transitions in proteins is a definition of a set of variables (reaction coordinates) connecting the multiple conformational states of the system. Often, such choices are based on a preconceived notion about tentative mechanism of the process. Several models that explain the mechanics for the transporter sliding from state to state have been proposed. The two most popular models are the rocking bundle model [31] and the flexing helices model [13], [14]. In the rocking bundle model [31], the rocking of a 4-helix “bundle” formed by TMs 1, 2, 6, and 7 against the “scaffold” formed by the rest of the essential TMs (TMs 3, 4, 5, 7, 8, 9, and 10) allows the transporter to alternatively open to either side of the membrane. In the flexing helices model [13], [14], flections in the two helices with a helix-break-helix motif in the middle (TM1 and TM6) of the TM section are proposed to be the major trigger for the conformational changes that allow alternating access. These two models are not necessarily mutually exclusive. The mechanics described in these models may work together to achieve the functions of Na-dependent secondary-active transporters [13], [16], [32], [33]. In a number of available crystal structures, the features of both models seem to be present, albeit to a different degree. For example, the flexing helices model is highly featured in LeuT [13] while the rocking bundle model is associated with apparent symmetry between different states of Mhp1 transporters [11], [13], and a mixture of both modes of transport was proposed to be essential for BetP [16].

**Figure 1:**
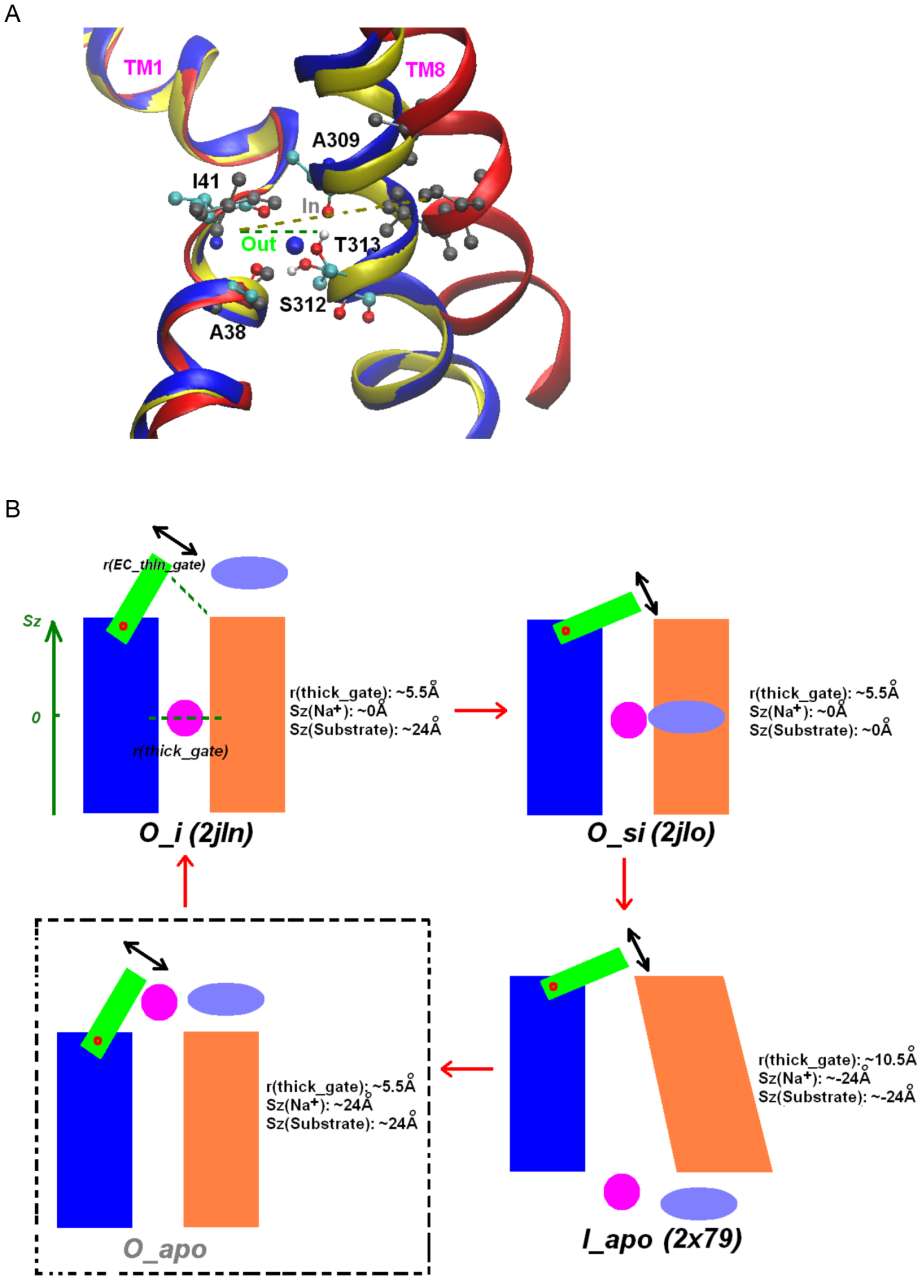
Order parameters and simplified illustration of a transport cycle. (A) Order parameter r(thick_gate). Overlap of the Na^+^ site for the outward-facing state with ion and substrate bound (*O_si*, pdb entry 2jlo, blue ribbon, blue ball for Na^+^, and ball-stick representation for Na^+^ coordinating residues), outward-facing state with ion bound (*O_i*, pdb entry 2jln, yellow ribbon), and the inward-facing apo state (*I_apo*, pdb entry 2×79, red ribbon, and grey ball-stick representation for coordinating groups). The order parameter is defined by the Center of Mass (COM) distance between the Na^+^ coordinating residues from TM1 (A38, I41) and TM8 (A309, S312, T313). The r(thick_gate) is at ∼5.5 Å in outward-facing states (*O*_*i*, *O_si*), comparing to ∼10.4 Å in the inward-facing open state (*I_apo*). (B) Schematic illustration of a transport cycle of the Mhp1 transporter. The transport cycle was simplified by 4 sequential conformational states: *O_apo*→*O_i*→*O_si*→*I_apo*. For each conformational state, the “bundle” and “hash motif” [11], [31] helices are represented in blue and orange shapes respectively. EC and IC thin gates are omitted for clarity. Na^+^ and the substrate are represented as a magenta circle and a purple oval respectively. Typical values of the order parameters for each state are listed beside its illustration.

Regardless of the gating model, natural reaction coordinate for ion-dependent conformational dynamics relevant to the process can be reduced to the distance between the groups of residues forming the conserved Na^+^ site (Na2 of LeuT) from the two TMs (TM1 and TM8). This conserved Na^+^ site sits between the interface of the “bundle” and “scaffold” of the rocking bundle model [31]. This site is also directly beneath the break in the TM1 helix that was shown to be critical in the flexing helix model [13]. Thus, following Shimamura *et al.* [11] suggestion, we use the Na2 site distance, defined as the distance between the center of mass (COM) of the Na^+^-coordinating residues in TM1 (Ala38, Ile41) and TM8 (Ala309, Ser312, and Thr313), as an order parameter to indicate the conformation of the transporter protein. Na2 ion binding has been strongly associated with the gating of the so-called “thick gate” [14]: ∼20 Å thick of compactly packed protein blocking the accessing path to the intracellular side for the substrate when it is closed. For clarity, we use r(thick_gate) to describe the order parameter. A r(thick_gate) of ∼5.5 Å indicates outward-facing state, whereas a r(thick_gate) of approximately 10 Å corresponds to inward-facing state (Figure 1).

In addition to the “thick” gate, an extracellular thin gate and an intracellular thin gate have been proposed for Mhp1 to explain the alternating access mechanism in a “modified” rocking bundle model [11]. In this model, Mhp1 changes orientation through the rocking of the “bundle” helices formed by TMs 1, 2, 6, and 7 relative to a reduced “scaffold”, or the so-called “hash motif” (helices of TMs 3, 4, 8, and 9) (Figure S1). When the protein faces outward, TM10 could serve as an extracellular thin gate and bend to block the EC vestibule. In contrast, when the protein faces inward, the proposed IC thin gate TM5 bends and opens the IC vestibule. To account for the movement of the EC thin gate, an additional reaction coordinate r(EC_thin_gate) is defined by the COM distance of the mobile TM9/TM10 loop residues (Asn360, Thr361, and Phe362) and the stationary TM1 residues (Ile47, Ala48, and Ala49) following ref. [11]. The dynamics of the IC thin gate has been shown to nearly synchronize with those of the thick gate [11] and thus is redundant for describing the reaction coordinate. The order parameters (Sz(Na^+^) and Sz(substrate) describing binding/release of Na^+^ and the benzyl hydantoin substrate are defined as vertical (Cartesian z-direction of the simulation system) positions relative to their respective binding sites. Thus, Sz around 0 Å corresponds to a location of the binding site, while a large positive and negative Sz value indicates that the ion/substrate is at the EC and IC side respectively. The exact definitions of the order parameters are provided in Table S1. Typical values of order parameters for various conformational states of Mhp1 are illustrated in Figure 1B.

### The minimal-energy transport cycle from simulations with a string method

We obtained a relaxed transition path for the entire transport cycle using the string method with swarm-of-trajectories [25], [26] (see methods). This relaxed path is represented by 134 intermediate images that connect the three conformational states. RMSD analysis (Figure S2) of the path shows that the modified rocking bundle model [11] can dynamically represent the entire cycle, consistent with an earlier report obtained from coarse-grained molecular dynamics [19]. Throughout the transport cycle, backbone RMSD changes within the “bundle” or the “hash motif” (Figure S1) were less than 1.2 Å, whereas backbone RMSD changes for the entire transporter were much higher, up to 3 Å (Figure S2). The structures corresponding to finally relaxed transport path were used for free energy simulations using umbrella sampling and WHAM.

### Release of Na^+^ leads the way for the transition from *O_si* to *I_apo*

**Figure 2:**
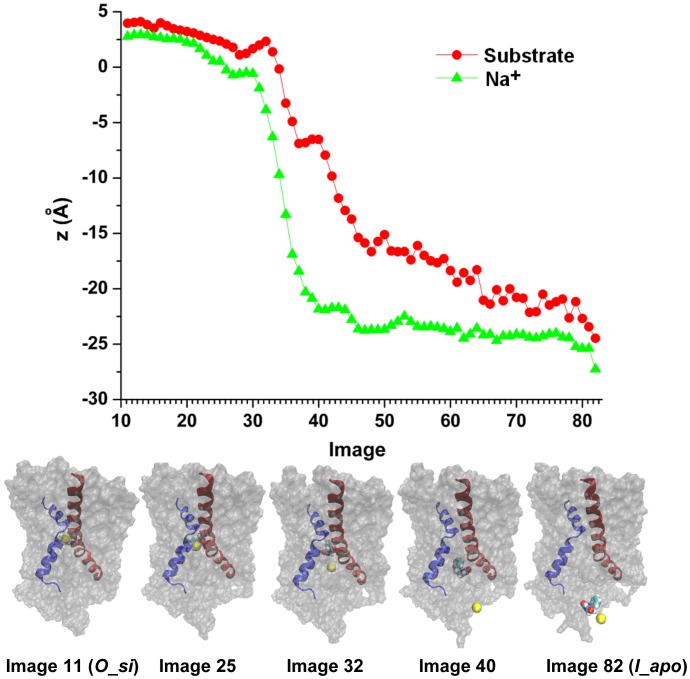
Sequence of Na^+^ and substrate release for the transition from occluded state with both Na^+^ and substrate bound (*O_si*) to inward-facing apo state (*I_apo*) from our path obtained with string method with swarm of trajectories. The path consists of 72 images with Image 11 being in *O_si* and Image 82 being in *I_apo*. In the top panel, the values of the z component of Cartesian coordinates as a function of the image number are shown for the Na^+^ (green triangles) and the substrate (red circles). In the bottom panel, initial, final and 3 intermediate images of the path are shown. TM1 (residue 23 to 55) and TM8 (residue 296–329) are shown in blue and red cartoons respectively, with the remaining of the Mhp1 protein shown in grey surface representation. Na^+^ is shown as a yellow ball and the substrate is shown as a collection of cyan, white, and red balls.

The causality in the mechanism of release of the ions and substrate from the occluded state of LeuT fold secondary transporters has been an intriguing topic [1], [17], [22], [32], [34]. It has been proposed that the binding of a substrate to the main substrate binding site labeled “S1” perturbs the Na^+^-binding site [32], promoting the release of Na^+^. Our computations [17] indicated that slow wetting from the intracellular side may also facilitate the release of Na^+^. Regardless of the driving forces, a large amount of evidence [17], [22], [24], [32], [34]–[36] indicates that the release of Na^+^ precedes the release of the main substrate. Our relaxed transition path from the string method strongly supports this notion. In Figure 2 we illustrate the path that describes transition from *O_si* to *I_apo*, represented with 70 intermediate images (Image 12–81) connecting the initial *O_si* (Image 11) and final *I_apo* (Image 82) states. Na^+^ starts moving towards the intracellular site first (∼Image 25), while the substrate remained bound even when ion has escaped from its binding site to ∼8 Å beneath the Na^+^ site of the occluded *O_si* state (∼Image 32).

**Figure 3:**
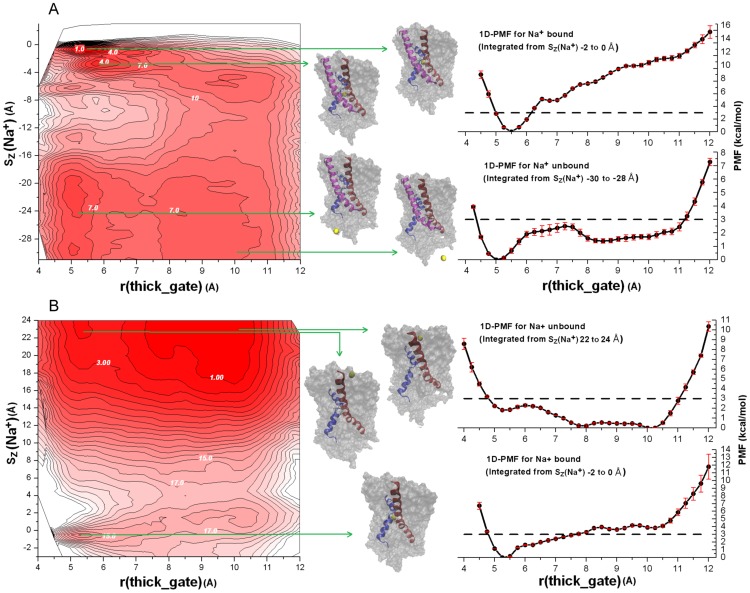
Coupling between Na^+^ binding and Mhp1 conformational state. Conformational distribution of the Mhp1 thick gate highlighting the transition between states of (A) outward-facing occluded (*O_si*) and inward-facing open (*I_apo*), and (B) inward-facing open (*I_apo*) and outward-facing open ion-bound(*O_i*). In the left panel, the free energy landscapes governing the open/closure of the thick gate (r(thick_gate)) as a function of the binding of the Na^+^ (Sz(Na^+^)) calculated from all-atom umbrella sampling MD simulations with explicit membrane and solvent are shown in 2D map. Each contour line corresponds to 1 kcal/mol. PMF value of selected contour lines is marked with white numbers. The landscapes vary from red to white with darker colors indicating more favorable conformation. In the centre panel, representative conformations are shown for the indicated regions in 2D PMF's. Na^+^ is shown in yellow ball while the residues forming Na2 site are shown in ball-and-stick. TM1 (residue 23 to 55) and TM8 (residue 296–329) are shown in blue and red ribbons respectively. For (A), TM5 (residue 160 to 192) that is more involved with Na2 ion dynamics is shown in purple ribbon. The rest of the protein is shown in grey surface representation. In the right panel, (A) 1D-PMF over the Mhp1 thick gate for Na^+^ bound (top panel) and Na^+^ unbound (bottom panel), integrated from Sz(Na^+^) of −2 to 0 Å and −30 to −28 Å of the left panel respectively. (B) 1D-PMF over the Mhp1 thick gate for Na^+^ unbound (top panel) and Na^+^ bound (bottom panel), integrated from Sz(Na^+^) of 22 to 24 Å and −2 to 0 Å of the left panel respectively. The dashed lines at 3 kcal/mol are placed for reference. Error bars are standard deviations computed by blocking the umbrella sampling data into three blocks.

Next we examined the free energy landscape underlying Na^+^ release from the transporter by calculating the 2D PMF for reaction coordinates that describe vertical (across the lipid bilayer) displacement of an ion relative to its binding site (Sz(Na^+^)) and the state of the thick gate (r(thick_gate)) (Figure 3A). The computed PMF shows that the initial stage of the Na^+^ release and the inward-facing opening of the thick gate, is a concerted process. 2D PMF for an ion release process (Figure 3A) shows a meta-stable Na^+^ binding site, denotes Na2', near [5.5 Å, −3.0 Å]. The relative difference in stability between the main binding site and the meta-stable site is ∼5 k_B_T. In the meta-stable site, Na^+^ is still coordinated by Ile38 of TM1 and Ser312 and Thr313 of TM8 but breaks away from A41 of TM1 and Ala309 of TM8 to coordinate to Asn168 of TM5 (Figure 3A, center panel, and Figure S3). Thus, Asn168 of TM5 may provide an attractive force that chaperones Na^+^ escape from Na2, similar to the role proposed for Asp189 in vSGLT [24], [32] and Glu192 in LeuT [37]. On the other hand, the interaction of TM5 residues to the Na+ bound to the Na2 site suggests that Na2 and Na2' may play an important role in regulating the dynamics of TM5. Which in turn may serve as the proposed intracellular thin gate of Mhp1, consistent with an earlier study on LeuT by Shi *et al.* [23]. Figure S3 (right panel) illustrates how TM5 and TM8 move and open up the intracellular pathway for the I_*apo* state, when Na^+^ is released providing further support for this mechanism.

### Binding of Na^+^ promotes the stabilization of outward-facing state

The PMF maps collected in Figure 3A (left panel) show essentially a flat energy surface with Na^+^ fully dissociated from the transporter (Sz(Na^+^) at −32 Å to −28 Å). There is only a small difference (<2 k_B_T) in the PMF values between the outward-facing states (r(thick_gate) at ∼5.5 Å) and the inward-facing states (r(thick_gate) at ∼10 Å). This result suggests that energy of a thermal bath is sufficient for stochastic shuttling of empty transporter between outward-facing and inward-facing states. In contrast, the binding of Na^+^ helps to stabilize the outward-facing state of the transporter, indicated by a favorable minima at a r(thick_gate) of ∼5.5 Å relative to a shallow well at ∼10 Å (inward-facing states of the system).

To highlight the coupling between ion binding and energetics of gating process we computed 1D PMF slice from 2D PMF map for the r(thick_gate) dynamics (Figure 3A, right panel). These 1D PMF profiles provide a measure for the relative stability of the outward-facing and inward-facing states. When a Na^+^ is bound, the outward-facing states (r(thick_gate) ∼5.5 Å) are ∼16 k_B_T more favorable than the inward-facing states (r(thick_gate) ∼10 Å). In contrast, when the Na^+^ is released, the PMF difference between the outward-facing and inward-facing states is within 2–3 k_B_T. To show that the stabilization of the outward-facing conformations in Figure 3A is mainly caused by the binding of Na^+^ but not the substrate, we computed 2D PMF maps (Figure 3B, left panel) for the transition from the inward-facing open (*I_apo*) state to the outward-facing state with a Na^+^ bound (*O_i*), for which a substrate is not involved. Similar to Figure 3A, the 2D PMF also shows that a bound Na^+^ leads to significant stabilization of outward-facing conformations. In addition, without a bound Na^+^, the PMF profile was essentially flat for the transition between the inward-facing (r(thick_gate) ∼10 Å) to the outward-facing (r(thick_gate) ∼5.5 Å) states with a barriers of ∼4 k_B_T (Figure 3B, upper right panel).

The small barrier between the inward-facing and outward-facing states when the Na^+^ is unbound suggests that the transition between these states is likely and can be driven by a thermal energy of the bath. This mode of gating is not uncommon in secondary transporters. For example, in an experimental study of LeuT gating using single-molecule fluorescence resonance energy transfer methods, outward-facing and inward-facing states were shown to be in equilibrium without Na^+^ binding [22]. Reyes *et al.* proposed relatively unobstructed (stochastic) gating for the ion/substrate-free Glt_Ph_ transporter from the EAAT family [38]. The binding of an ion and a substrate merely shifts the stability of the system towards one of the states, whereas the empty protein can shuttle up and down the membrane [38].

### Substrate binding leads to the closure of the EC gate and a transition from *O_i* to *O_si*

**Figure 4:**
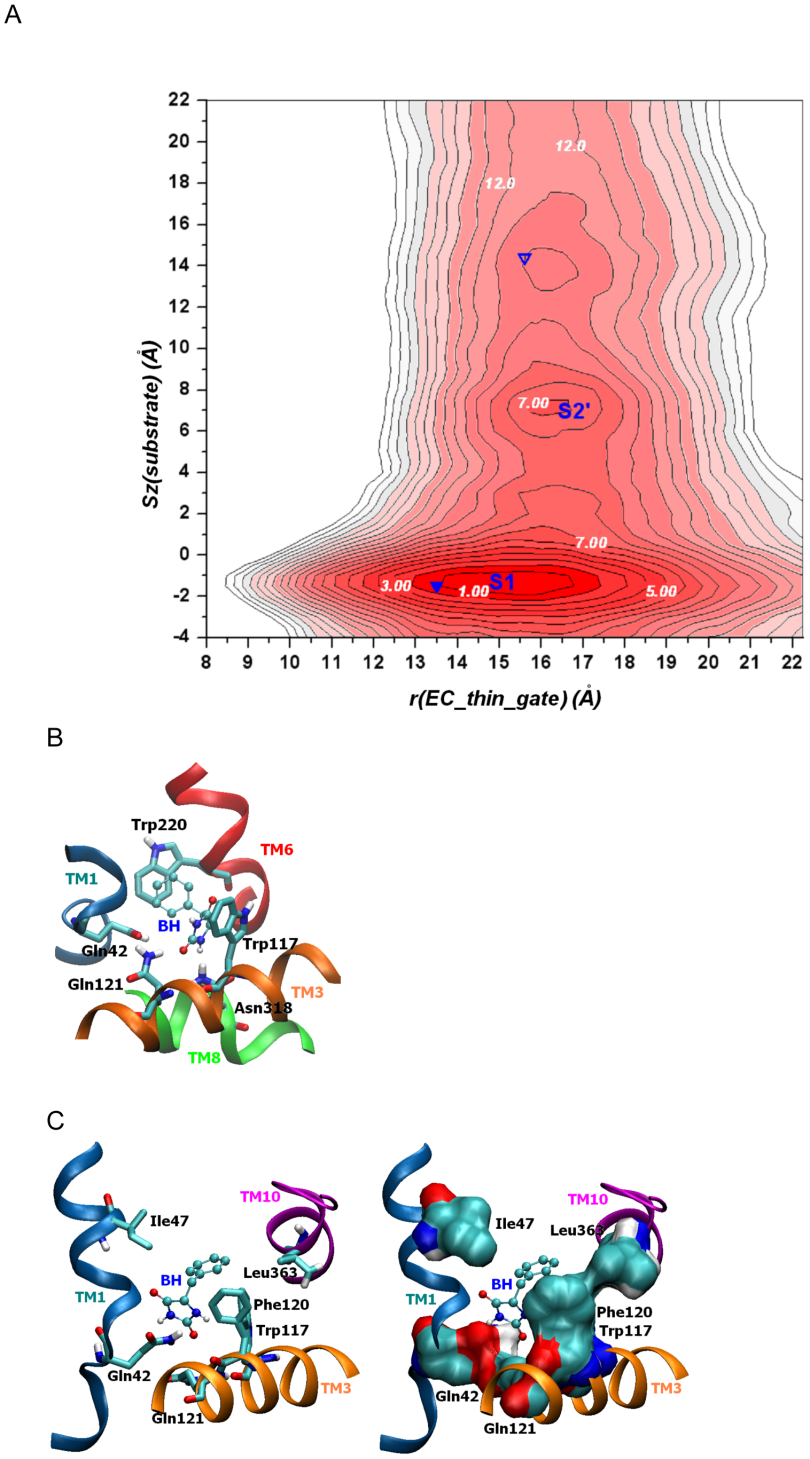
Coupling between the Mhp1 EC thin gate and substrate binding. (A) The free energy landscapes governing the open/closure of the EC thin gate r(EC_thin_gate) during the binding process of the substrate (Sz(substrate)) calculated from all-atom umbrella sampling MD simulations with explicit membrane and solvent. Each contour line corresponds to 1 kcal/mol. PMF value of selected contour lines is marked with white numbers. The landscapes vary from red to white with darker colors indicating more favorable conformation. The conformations from equilibrium MD for the outward-facing open state (*O_i*) and outward-facing occluded state (*O_si*) are projected on the map as an open inverted triangle and a filled inverted triangle respectively. (B) A snapshot of the substrate (benzyl-hydantoin, or BH, in ball-and-stick representation) bound in the primary S1 binding site. TM's 1, 3, 6, and 8 are shown in ribbons with major coordinating groups highlighted in sticks. (C) A snapshot of the BH substrate in the putative S2' binding site. In the left panel, TM's 1, 3, and 10 are shown in ribbons with major coordinating groups highlighted. In the right panel, surface representation is used to map major coordinating groups. The substrate has hydrogen bond and strong electrostatic interactions with Gln42 and Gln121 and stabilizing non-polar interactions with Ile47, Phe120, and Leu363.

The transition from the outward-facing Na^+^-bound *O_i* state to the outward-facing occluded state with binding of both Na^+^ and the substrate (*O_si*) features the binding of the substrate from the extracellular bulk to the primary S1 substrate binding site and the occlusion of the S1 site through the closure of the EC thin gate [11]. We quantified the free energy landscape of this transition by computing the 2D PMF map (Figure 4A) with two reaction coordinates describing dynamics of the EC thin gate (r(EC_thin_gate)) and substrate binding (Sz(substrate)). The PMF map indicates a global minimum for the substrate at a Sz(substrate) of approximately −2 to −1 Å. This minimum corresponds to the primary substrate binding site (S1) of the occluded Mhp1 (*O_si*), in which the substrate forms hydrogen bonds with Asn318 (TM8) and Gln121 (TM3) and aromatic ring-ring interactions with Trp117 (TM3) and Trp220 (TM6) (Figure 4B, left panel). At this minimum, the EC thin gate is relatively flexible as indicated by the small barriers featured in the PMF map (<5 k_B_T) for the gating distance transition from 12 Å (relatively closed) to 18 Å (widely open).

A second minimum (S2') is found at the Sz(substrate) position of ∼7 Å, approximately 8–9 Å above the S1 substrate binding site. In addition to residues from TMs 1 and 3 (Gln42, Ile47, Phe120, and Gln121), this site also involves residues from TM10 (Leu363), consistent with the putative S2 sites proposed for LeuT [22], [39] and DAT [40]. This suggests that S2 may be involved in a substrate entrance providing a low-affinity binding site. However, we note that this second free energy minimum (S2) for substrate is less favorable than in S1, with relative difference in stabilization of about 10 k_B_T. From Figure 4A, the optimal sequence for the transition from the *O_i* state to the *O_si* state is as follows. The substrate binds to the substrate binding site (S1) from the EC bulk, potentially through the putative S2' site during the transition. The EC thin gate then closes to occlude the substrate from the EC bulk. However, the EC thin gate displays significant conformational flexibility for this state as reflected by the flat PMF landscape for the opening/closure of the EC thin gate. Overall, the binding of a substrate to the S1 site for an *O_i* state transporter is an energetically favorable process. The PMF map shows a PMF drop of ∼16 k_B_T for the substrate binding to S1 relative to be in the bulk, suggesting mM affinity.

The data collected is insufficient to provide a definitive answer whenever S2 plays any role in conformational dynamics of the transporter. Shi et al. [22], [39] proposed a mechanism in which allosteric regulation mechanism of LeuT gating requires an existence of optimal conformational state (for S2 binding) at which substrate in S2 can lead to conformational switching and it is one of many possible combinations of ion/substrate loads. Therefore complete evaluation of conformational switching due to substrate binding to S1 and S2 would require full evaluation of multiple energy surfaces as function of substrate and ion loads for every stable minima of the system and is computational unfeasible at the moment.

### Coupling between Na^+^ and the substrate: Substrate binding restricts the release of Na^+^ back to extracellular bulk

Our results highlight the role of Na^+^ in the transport cycle: the binding of Na^+^ promotes the outward-facing conformation, and the release of Na^+^ allows the transition to an inward-facing conformation. Therefore, the binding of Na^+^ is coupled to the binding of the substrate by preparing more Mhp1 protein in the outward-facing conformation, to which the substrate can readily bind. On the other hand, the binding of the substrate was also shown to promote Na^+^ binding [11]. Our free energy profiles for the transport of Na^+^ and substrate provide the molecular underpinning of this coupling mechanism.

**Figure 5:**
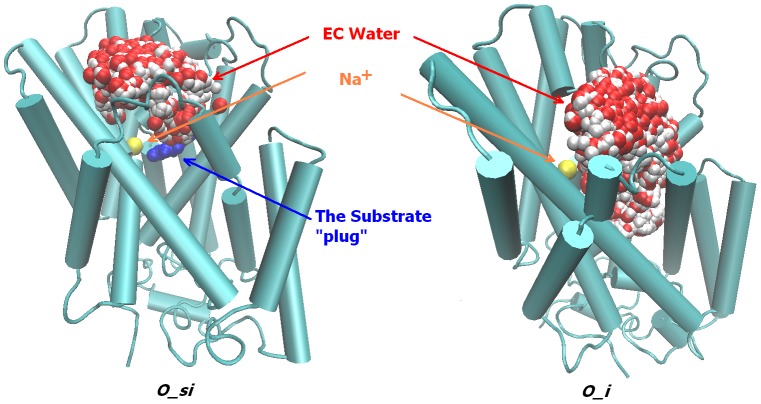
Access to EC bulk for Na2 ion in *O_si* and *O_i* states of Mhp1. Frames for equilibrium MD simulations of the *O_si* and *O_i* states of Mhp1 are shown. Protein (in blue cartoon), Na2 ion (in yellow ball), and the substrate (in blue ball) are shown just the first frame while EC vestibule water molecules (in red and white balls) are shown for 60 frames of the MD simulations.

In particular, Figure 3B shows that binding of Na^+^ to an apo-state of Mhp1 in the absence of substrate is energetically unfavorable. The dehydration of a Na^+^ ion upon transfer from the extracellular milieu to a binding site (Figure 3B) in the *O_i* state is marginally unfavorable for the equi-molar IC/EC solutions used for the PMF computations. However, the small barrier for an ion to escape to the extra-cellular milieu suggests rapid ion exchanges with the bulk (Figure 3B). At the same time, the binding of the substrate to the *O_i* state (Figure 4A.) is an energetically favorable process that aids the stabilization of a protein in the *O_si* state. The presence of a bound substrate obstructs the unbinding and escape of Na^+^ to the extracellular milieu. Figure 5 suggests that the bound substrate serves as a plug to restrict the release of Na^+^ to the extracellular bulk [11]. As a result, once locked in an occluded state (*O_si*), Na^+^ is driven to unbind into the intracellular solution.

### Note on the energetics of the entire transport cycle

The simulations shows that the Mhp1 transport cycle complies well with the postulated steps within an alternating access mechanism [2], [10] i.e.

1. The main substrate is translocated across the lipid membrane against its concentration gradient;
2. The coupled Na^+^ is translocated across the lipid membrane down its concentration gradient; and
3. The transporter protein serves as a catalyst and changes back to its original state.

**Figure 6:**
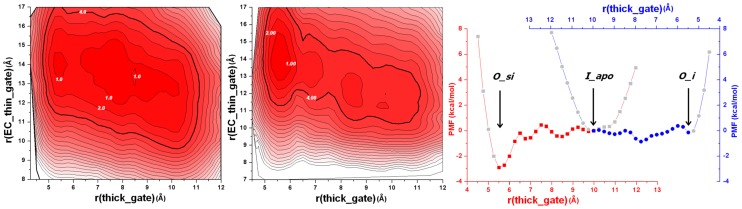
Relationship between conformational stability and gating dynamics. In the left and centre panels, 2D-PMF profiles on the thick gate (r(thick_gate)) and EC thin gate (r(EC_thin_gate)) are calculated from all-atom umbrella sampling MD simulations with explicit membrane and solvent. The transition between outward-facing state with the ion and substrate bound (*O_si*) and inward-facing open (*I_apo*) and between inward-facing open (*I_apo*) and outward-facing with only the ion bound (*O_i*) are shown in the left and centre panel respectively. The landscapes vary from red to white with darker colors indicating more favorable conformation. Each contour line corresponds to 1 kcal/mol. PMF value of selected contour lines are marked with white numbers. The areas that are within 4 kcal/mol (<7 k_B_T) are circled by the darker contours. In the right panel, 1D PMF profiles integrated from the left and centre panels are shown as red squares and blue circles (red and blue X-coordinate correspond to the red and blue profile, respectively). The profiles represent the stability of the transporter as a function of the r(thick_gate) during the two transitions. The two profiles are placed side to side with an overlap around the *I_apo* state, which is set to 0 for PMF, so that they provide an overall stability picture for the transitions from *O_si* (r(thick_gate) ∼5.3 Å in crystal structure, red) to *I_apo* (r(thick_gate) ∼10.2 Å in crystal structure, red and blue) to *O_i* (r(thick_gate) ∼5.4 Å in crystal structure, blue) [11], [12].

The analysis of multiple PMFs allows for understanding of energy flow and coupling between different stages in a transport cycle (summarized in Figure 6). Starting from the outward-facing state with Na^+^ bound (*O_i*), the substrate binds to the primary substrate-binding site (S1), facilitating formation of the *O_si* state. The binding of Na^+^ and a substrate results in the closure of the extracellular (EC) thin gate and the blockage of Na^+^ unbinding to the EC solution. In the next step of the transition, Na^+^ unbinds to the intracellular (IC) side [17], [22], [24], [32], [34]–[36], and the transporter protein opens its thick gate towards the IC side in a concerted movement. The substrate is then released to the IC solution. It is important to note that releases of Na^+^ (Figure 3A) and the substrate (Figure S4) are energetically unfavorable. With the release of the Na^+^ and the substrate, the Mhp1 transporter is in the inward-facing apo state (*I_apo*). To reset the transport cycle, the protein is required to change its conformation back to the outward-facing state. Little is known about the driving force for this step [22], [38], [41]. Our 2D PMF computations (Figure 3B and Figure 6) show that for an empty transporter (both ion and a substrate are released) the energetic barriers separating each of the conformational states are marginal. The transporter can shuttle back and forth until the binding even of a Na^+^ to Mhp1 promotes the stabilization of the outward-facing state *O_i*. PMF shows that *I_apo* and *O_apo* states separated by a very small energy barrier. This result is consistent with the mechanistic principles proposed earlier by Gouaux et al. (Figure 3 of Ref. [18]). One unresolved feature of the above mechanism is with the identification of key driving forces responsible for Na^+^ binding and subsequent release from the Mhp1 protein. In addition to Asn168, which may facilitate the release of Na^+^, we propose that *local* concentration gradients and a partial surface hydration might play an important role [22] in this process. At the *O_si* state, the local Na^+^ concentration in the Na^+^ binding state is very high relative to the bulk phase because of the small volume of the Na^+^ site providing both attractive pathway for slow hydration and a driving force for ion release.

### Conclusions

In review, the free energy landscapes collected from over 4 µs of all-atom MD simulations predict that Na^+^ binding drives the cycle by promoting outward-facing states of Mhp1, thus preparing conformations for the subsequent substrate binding. In contrast, the substrate mainly improves the apparent binding affinity of Na^+^ by restricting the release of Na^+^ back to the EC bulk. These computational results explain the experimental results on coupling between Na^+^ and benzyl-hydantoin binding to Mhp1 [12]. Our results also indicate that Asn168 of TM5 plays an important role in Na^+^ release to the IC bulk, reminiscent of the role assigned to Asp189 in vSGLT transporters [24], [32] and Glu192 in LeuT [37]. This result supports the findings reported for LeuT and ApcT transporters [23] where Na2 ion was proposed to be essential for modulation of the IC thin gate. It was found that barriers separating an inward-facing apo and an outward-facing apo states are very low (2–4 k_B_T only). This result is consistent with experimental study of LeuT [22] and the proposed stochastic shuttling for the Na-dependent secondary transporter glutamate transporter Glt_ph_ [38]. Therefore, the low-barrier shuttling of empty proteins appears to be general feature in many secondary transporter systems.

## Methods

### MD simulation

Structures of the Mhp1 transporter [11], [12] in the states of outward-facing with Na^+^ bound (*O_i*, pdb entry 2jln), outward-facing occluded with both the substrate and Na^+^ bound (*O_si*, pdb entry 2jlo), and inward-facing apo (*I_apo*, pdb entry 2×79) were taken from the X-ray coordinates stored in the Protein Data Bank. The simulation system was built using the web-based CHARMM-GUI membrane builder [42], containing one Mhp1 transporter, Na^+^ and the substrate when present, and 150 lipids molecules with a native-like 4∶1 mixture [11] of 1-palmitoyl-2-oleoyl phosphatidylethanolamine (POPE) and 1-palmitoyl-2-oleoylglycero-3-phosphoglycerol (POPG) solvated by 100 mM NaCl in an aqueous solution. The full simulation cells include ∼60,000 atoms in a hexagonal box with a dimension of approximately 43 Å in edge length and 94 Å in height (Figure S5). The MD simulations were carried out by CHARMM version c36a2 [43] using the CHARMM27 force fields with cross term map (CMAP) corrections for proteins and lipids [44]. The TIP3P model was used for water molecules [45]. The force field parameters for the benzyl-hydantoin (BH) substrate (Dataset S1) were generated using the paramchem web-based interface [42] which applies the philosophy of the CGenFF force field [46]. A constant pressure/temperature (NPT) ensemble was used for all simulations with a pressure of 1 atm and a temperature of 298.15 K with the Nose-Hoover thermostat [47], [48]. Long-range electrostatic interactions were calculated using particle mesh Ewald (PME) algorithm [49] with a 96 Å by 96 Å by 108 Å grid for a fast Fourier transform. A non-bonded interactions switching function over 12 to 16 Å was used in all MD simulations. The leap-frog algorithm was applied to integrate the equation of motion with a time step of 2 fs. Following a staged equilibration with a gradual decrease in harmonic constraints acting on heavy protein atoms, a further equilibration was run for 10 ns without any configurational constraints. These equilibrated systems were then used to perform targeted molecular dynamics simulations.

### Targeted molecular dynamics simulation

The TMD module [50] in CHARMM [43] was applied for targeted molecular dynamics simulations. Three transitions were simulated: *O_si* to *O_i*, *O_si* to *I_apo*, and *I_apo* to *O_i*. To match the state of *O_si*, A substrate was placed in the extracellular vestibule (∼13 Å above the substrate binding site) to the *O_i* state and a substrate and a Na^+^ were placed in the intracellular vestibule (∼26 Å below the substrate binding site) to the *I_apo* state. As the EC and IC vestibules in those respective states are open to the EC and IC bulk, the substrate and Na^+^ can diffuse to the bulk easily, i.e., they are in a relatively flat free energy surface. Thus, the exact placement of the missing substrate and Na^+^ to *O_i* and *I_apo* states shall not affect the optimal transition paths. For each transition, the initial state was slowly pulled to the targeted state by applying a harmonic restraint to reduce the RMSD between the two states. The selection of atoms for which the RMSD constraint is applied includes the protein heavy atoms, and the ligand Na^+^ and the 5′ carbon (Dataset S1) of the hydantoin group in the substrate. The speed for RMSD constraint evolution was set at 0.00005 Å/step. The simulation was terminated when the system was within a RMSD difference of 0.02 Å to the target.

### Transition path sampling using the string method with swarm of trajectories

Large-scale conformational changes in biomolecules, such as those involved in the transport cycle of Mhp1, are complex processes taking place on timescales that can be well beyond the limit of brute force molecular dynamics simulations [26]. In this work, we applied the recently developed and emerging string method with swarm of trajectories [25], [26], [51]–[54] to obtain optimal paths connecting the structurally available conformational states of the Mhp1 transporter. The string method aims to find the minimum free energy path (MFEP) in the subspace of a large but finite set of coordinates, **z**, referred to as “collective variables” [52]. A path is ordered as a chain of *M* states or “images”, connecting two stable conformations. In this work, we explore the transition paths between the Mhp1 conformational states of *O_i*, *O_si*, and *I_apo*. These paths complete the transport cycle. To study the conformational transitions of the transporter and the translocation of the substrate and Na^+^, the collective variables include the Cartesian coordinates of the transporter backbone atoms, Na^+^, and 5′ carbon of 5-benzyl hydantoin (C5). We only included one atom (C5) in the collective variables for the substrate in order to avoid an orientational bias to the substrate. The initial paths were obtained from targeted molecular dynamics [50] as described above. There are 9, 70, and 55 intermediate states, or images, for the path connecting *O_i* to *O_si*, *O_si* to *I_apo*, and *I_apo* to *O_i* respectively. The numbers of intermediate states were chosen so that the average RMSD of the collective variables between adjacent images is less than 0.2 Å. For each image, the simulation system has 59775 atoms, including the Mhp1 protein, the substrate, the Na^+^, lipid molecules, water, and Na^+^ and Cl^−^ counter ions. Therefore, the effect of lipid bilayers and water were taken into account explicitly.

The iteration of the string method generally followed the procedures by Gan et al. [25] using NAMD [55] and VMD [56]. Each iteration consists of 4 steps: generation of the swarm of trajectories, evolution of the image, run of constraint MD, and re-parameterization. First, for each image, 100 2-ps-long MD simulations were carried out. Second, the coordinates of the collective variables for the 100 trajectories were averaged. Third, 50 ps of MD simulations were then carried out with a strong harmonic constraint (40 kcal/mol Å) on the collective variables to evolve the collective variables to the average drift and relax the rest of the system other than the collective variables. Finally, the images are re-parametrized to ensure that they are evenly distributed in terms of collective variables along the new path, which, in our case, means the RMSD difference of the atoms in the set of collective variables between adjacent images are roughly equal. This final step ensures that the images are not trapped in local minima.

Following Ovchinnikov *et al.* [54], convergence of the string to the optimal path is evaluated each iteration by monitoring the average RMSD each image has moved from its initial conformation (solid red line in Figure S6). In addition, we also monitored the average RMSD each image has moved from the same image 4 iterations before (dotted blue line, Figure S5). Convergence is assumed when both lines reach a plateau. The transition paths are converged for *O_i* to *O_si*, *O_si* to *I_apo*, and *I_apo* to *O_i* after 30, 48, and 22 iterations. The three paths complete a transport cycle for Mhp1 and provide 137 energetically-relaxed images for the entire transport cycle. Structures from the path were chosen as appropriate starting structures for umbrella sampling simulations.

### Umbrella sampling and WHAM analysis

Umbrella sampling simulations were carried out using the NAMD program [55]. Harmonic constraints were applied to the order parameters using the collective variable module (colvars). Multiple windows were applied to cover the regions of interest. The window size for order parameter r(thick_gate) was 0.25 Å, while the order parameter r(EC_thin_gate), Sz(Na^+^), and Sz(substrate) were mapped with a window size of 0.5 Å (Table S1). The force constants were chosen to be 5 kcal/mol to ensure overlapping of the sampling of windows. Each window was sampled for 560 ps of MD simulation, and the last 500 ps of data were used for weighted histogram analysis [28]. Thus, each 2D-PMF was produced from a total simulation time of 0.8–2 µs. WHAM program [57] was used to obtain the 2D PMF profiles from the umbrella sampling data. The bin size was set at 0.25 Å, and the convergence tolerance was set at 0.001 kcal/mol. The 1D-PMF's were integrated from interested regions of the 2D-PMF's following a method used by Allen *et al.* [27]. The statistical uncertainties were estimated by blocking the data into three blocks.

Proper starting conformation for each window subject to umbrella sampling is essential for efficient computation of the PMF's. In the Mhp1 transport cycle, the conformational changes are too large for the allowance of a single starting conformation, because very long simulations are required to relax the protein structure to a specific window of the order parameters. For example, while the biased potential applied in umbrella sampling could force r(thick_gate) to a value around 8.0, from either outward-facing (r(thick_gate) ∼5.5 Å)) or inward-facing (r(thick_gate) ∼10 Å) structure, extremely long simulation time may be required to relax the rest of the protein. To avoid excessive long relaxation time, targeted molecular dynamics has been applied to obtain intermediate structures for use as starting structures for umbrella sampling [58]. In this study, we use the string method with swarm of trajectories to further relax the path from TMD simulations. To get a starting configuration for a specific window, we pick from the string-method path an intermediate configuration that has similar order parameter values for that specific window. When only one of two order parameters can be matched, priority was put on the one that usually needs more time to relax. For example, when no intermediate image has both order parameters close enough to a window centered at (r(thick_gate) = 8.0 Å, Sz(Na^+^) = −1 Å), we'd pick a intermediate image with r(thick_gate) around 8 Å, and let the harmonic constraint in umbrella sampling move Sz(Na^+^) to its designated value (Figure 6). For instance, in our computation for the 2D-PMF shown in Figure 3A, we used image 32 of from the transition path obtained from swarm-of-trajectories for this window (Figure S7). The absence of Na+ contamination for constructed apo-states was confirmed for each window of the umbrella simulations by running minimal contact distance analysis (Figure S8).

## Supporting Information

Dataset S1 Topology and Parameters for molecular simulations of L-5-Benzyl Hydantoin (L5BH).
(DOC)

Figure S1 Topology of Mhp1. The “bundle” helices (TM's 1,2,6,7) are shown in red and blue. The “hash motif” (TM's 3,4,8,9) helices are shown in orange. They form a hash “#” sign and thus the name. Na2 ion is shown in magenta. The site is formed by TM1 and TM8, at the interface of the “bundle” and the “hash motif”. The substrate binding site is illustrated as blue oval [11], [31].
(TIF)

Figure S2 RMSD changes along the transition path from string method with swarm of trajectories. The RMSD values for the backbone heavy atoms of the residues between each image on the path and the reference image (*O_i*) are computed after a RMSD best-fit of the residues in selection. The RMSD values for residues ARG10 to GLY470 (All), the bundle (TM's 1,2,6,7), the hash motif (TM's 3,4,8,9), TM5, and TM10 are plotted in black squares, red circles, blue triangles, green stars, and purple crosses respectively.
(TIF)

Figure S3 The Na2 (left panel) and the proposed Na2' (centre panel) sites and its effect on inward-facing gating. Structures are taken from umbrella sampling simulations. The left panel represents a typical conformation (Na^+^ in Na2) at the global minimum in Fig. 3A while the centre panel represents a typical conformation at the nearby minimum when the Na^+^ moves down by ∼2 Å, coordinated by Asn168 of TM5 (Na^+^ in Na2'). In the right panel, the effect of Na bound and unbound on the intracellular gating is shown by comparing the *O_si* (blue) and *I_apo* (red) structure. In the *I_apo* structure, TM8 and TM5 move relatively away from TM1 to open up the ion release pathway to the IC bulk.
(TIF)

Figure S4 Releasing of the substrate from occluded state (O_*si*). Conformational distribution of the Mhp1 thick gate highlighting the transition between outward-facing occluded (O_*si*) and inward-facing open (*I_apo*). The figure depicts the potential of mean force landscapes governing the open/closure of the thick gate (r(thick_gate)) as a function of the release of the substrate (Sz(substrate)) calculated from all-atom umbrella sampling MD simulations with explicit membrane and solvent. Each contour line corresponds to 2 kcal/mol. PMF value of selected contour lines are marked with white numbers.
(TIF)

Figure S5 Simulation cell for the outward-facing occluded Mhp1 with both the Na^+^ (shown as a big yellow ball) and substrate bound (shown as a collection of cyan, white, red, and blue balls). The protein is shown in red tubes. Lipids POPE (grey) and POPG (cyan) are shown in lines. Water molecules, Na^+^, and Cl^−^ counter ions are shown in red lines, small yellow balls, and small cyan balls respectively. All molecular figures, including this one here, are generated with VMD [56].
(TIF)

Figure S6 Convergence of the swarm-of-trajectories string method transition path. Average RMSD between the images in the current iteration (iter_i) and their corresponding images in the initial path (iteration 0) as a function of iteration number is plotted in black squares for the transition of *O_si* to *I_apo*. Average RMSD between the images in the current iteration (iter_i) and their corresponding images 4 iterations before (iteration i-4) as a function of iteration number is plotted in black squares. Both lines approaches plateaus and convergence is assumed [54].
(TIF)

Figure S7 Mapping of the transition path obtained from string method to the 2D PMF about Sz(Na+) and r(thick_gate_distance) for the transition of *O_si* to *I_apo* and the illustration for selection of starting conformation for umbrella sampling simulations. The background 2D-PMF shows the free energy landscapes governing the open/closure of the thick gate (r(thick_gate)) as a function of the binding of the Na^+^ (Sz(Na^+^)) (Figure 3A). Each contour line corresponds to 1 kcal/mol. PMF value of selected contour lines is marked with white numbers. The landscapes vary from red to white with darker colors indicating more favorable conformation. The transition path from string method is projected to the 2D map with each intermediate structure shown with an orange number on the 2D map (number 11–82 represent the initial Image 11 to final Image 82). The orange numbers above the 2D map indicate the selection of the conformational structure for the umbrella sampling for sampling windows sit between the two green vertical lines. For example, for the umbrella sampling windows between r(thick_gate) 3.875 Å and 5.125 Å, Image 11 is used as the starting conformation.
(TIF)

Figure S8 The distribution of minimal distances between Na^+^ ions present in the system and any atom of the residues (A38, I41, A309, S312, T313) forming the sodium binding site for each (2-dimensional) window computed from the trajectories of the umbrella sampling simulations. The minimal distance distributions are from 7 Å to 32 Å for all the windows of umbrella sampling simulations leading to the 2D-PMF in Figure 3A. Na+ are not contaminating apo-state simulations.
(TIF)

Table S1 Definition of order parameters used in this paper. “COM” stands for Center Of Mass. z() represents the z component of the Cartesian coordinates.
(DOC)
