# Supplementary material for: The Molecular Mechanism of Ion-Dependent Gating in Secondary Transporters
Source: PLoS Comput Biol. 2013 Oct 24;9(10):e1003296. doi: 10.1371/journal.pcbi.1003296 (PMC3812048; doi:10.1371/journal.pcbi.1003296)
Supplement: Dataset S1 — Topology and Parameters for molecular simulations of L-5-Benzyl Hydantoin (L5BH). (DOC) [file pcbi.1003296.s001.doc]

!!! Topology and Parameters for Ligand L-5-Benzyl Hydantoin (L5BH)

!!! Nonbonding parameters are from CHARMM force field(Vanommeslaeghe et al. 2010)

!!! Point charges are obtained through restrained fitting to the quantum-mechanical electraostatical potentials (ESP) calculated at the B3LYP/6-31G* levels using the method outlined in ref. (Anisimov et al. 2005).

!!! Illustration for the chemical structure of 5-Benzyl Hydantoin


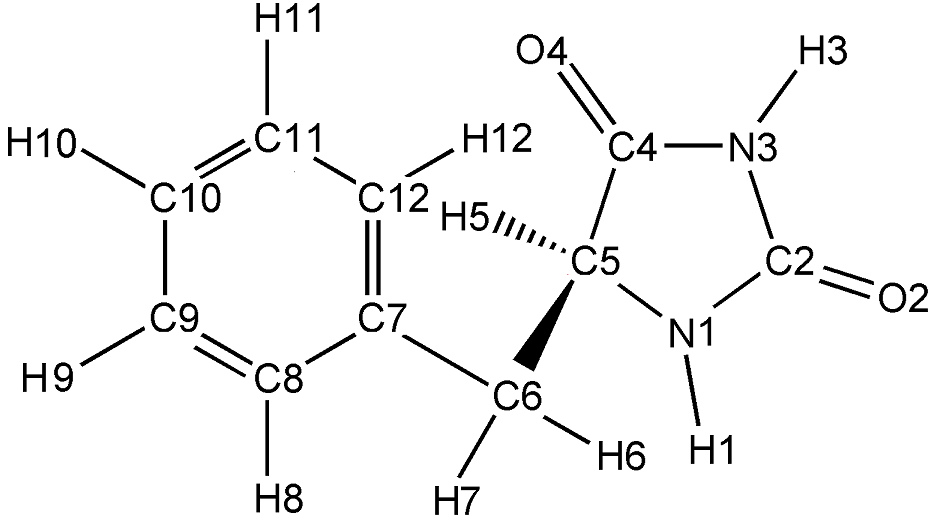


!!! CHARMM Format Topology File

RESI L5BH 0.00 ! L5BH is the residue name designated for L-5-Benzyl Hydantoin

GROUP

ATOM N1 NG2R -0.444

ATOM H1 HGP1 0.217

ATOM C2 CG2R 0.342

ATOM O2 OG2D -0.214

ATOM N3 NG2R -0.381

ATOM H3 HGP1 0.381

ATOM C4 CG2R 0.338

ATOM O4 OG2D -0.227

ATOM C5 CG3C -0.086

ATOM H5 HGA1 0.101

GROUP

ATOM C6 CT2 -0.161

ATOM H6 HA 0.112

ATOM H7 HA 0.112

GROUP

ATOM C7 CA 0.029

ATOM C8 CA -0.100

ATOM H8 HP 0.112

ATOM C9 CA -0.122

ATOM H9 HP 0.080

ATOM C10 CA -0.131

ATOM H10 HP 0.072

ATOM C11 CA -0.122

ATOM H11 HP 0.080

ATOM C12 CA -0.100

ATOM H12 HP 0.112

BOND N1 C5 N1 H1 N1 C2

BOND C2 N3

BOND N3 H3 N3 C4

BOND C4 C5

BOND C5 H5 C5 C6

BOND C6 H6 C6 H7 C6 C7

BOND C7 C8

BOND C8 H8

BOND C9 H9 C9 C10

BOND C10 H10

BOND C11 H11 C11 C12

BOND C12 H12

DOUBLE C2 O2

DOUBLE C4 O4

DOUBLE C7 C12 C8 C9 C10 C11

DONOR H1 N1

DONOR H3 N3

ACCEPTOR O2 C2

ACCEPTOR O4 C4

IMPR C2 N3 N1 O2

IMPR C4 C5 N3 O4

IMPR C7 C6 C12 C8

PATCHING FIRS NONE LAST NONE

END

!!! CHARMM Format Parameter File

!!! Bonds

NG2R CG3C 370.0 1.45000 ! Analogy from NG2R CG3C52

NG2R HGP1 470.0 1.0150 !Analogy from 2PDO, 2-pyrrolidinone

NG2R CG2R 460.00 1.38000 !Analogy from 2PDO, 2-pyrrolidinone

CG2R CG3C 300.00 1.5300 ! Analogy from 2PDO, 2-pyrrolidinone

CG3C HGA1 307.00 1.1000 ! Analogy from THF, THF neutron diffr., 5/30/06, viv

CG3C CT2 222.50 1.5280 ! Analogy from TF2M, viv

CG2R OG2D 570.00 1.2350 !Analogy from 2PDO, 2-pyrrolidinone

!!! Angles

HGP1 NG2R CG2R 38.00 119.50 !

HGP1 NG2R CG3C 38.00 116.00 !

CG2R NG2R CG3C 75.00 111.00 ! 2PDO, 2-pyrrolidinone C5-N1-C2 v

NG2R CG2R OG2D 65.00 127.80 ! 2PDO, 2-pyrrolidinone

NG2R CG2R NG2R 120.00 105.50 ! 2PDO, 2-pyrrolidinone N1-C2-C3 v

CG2R NG2R CG2R 75.00 111.00 !

NG2R CG2R CG3C 120.00 105.50 ! 2PDO, 2-pyrrolidinone N1-C2-C3 v

OG2D CG2R CG3C 65.00 126.70 ! 2PDO, 2-pyrrolidinone

NG2R CG3C CG2R 140.00 113.70 ! NA

NG2R CG3C HGA1 43.00 111.00 ! NA From HGA1 CG3C NN2

NG2R CG3C CT2 140.00 113.70 ! NA

CG2R CG3C HGA1 34.50 110.10 22.53 2.17900 ! PROT alkane update, adm

CG2R CG3C CT2 58.00 109.50 11.16 2.561 ! THF, nucleotide CSD/NDB

! survey, 05/30/06, viv

HGA1 CG3C CT2 35.00 111.40 22.53 2.179 ! TF2M, viv

CG3C CT2 HA 33.430 110.10 22.53 2.17900 ! ALLOW ALI

! alkane frequencies (MJF), alkane geometries (SF)

CG3C CT2 CA 51.800 107.5000 !CT1 CT2 CA ALLOW ALI ARO

!!! Dihedral torsion

NG2R CG3C CG2R NG2R 1.600 3 0.00

NG2R CG3C CG2R OG2D 1.600 3 0.00

HGP1 NG2R CG3C CG2R 0.080 3 0.00

CG2R NG2R CG3C CG2R 1.600 3 0.00

NG2R CG3C CT2 HA 0.4000 3 0.00 ! par22, X CT1 CT2 X; erh 3/08

NG2R CG3C CT2 CA 1.6000 3 0.00 ! par22, X CT1 CT2 X; erh 3/08

HGP1 NG2R CG2R OG2D 0.8600 2 180.00 ! 2PDO, 2-pyrrolidinone

HGP1 NG2R CG2R NG2R 1.2700 2 180.00 ! 2PDO, 2-pyrrolidinone

HGP1 NG2R CG3C HGA1 0.0000 3 180.00 != 2PDO, 2-pyrrolidinone

HGP1 NG2R CG3C CT2 0.0800 3 0.00 ! 2PDO, 2-pyrrolidinone

CG2R NG2R CG3C HGA1 0.0800 3 0.00 ! 2PDO, 2-pyrrolidinone

CG2R NG2R CG3C CT2 2.3100 3 0.00 ! 2PDO, 2-pyrrolidinone

CG2R NG2R CG2R OG2D 2.5900 2 180.00 ! 2PDO, 2-pyrrolidinone

CG2R NG2R CG2R CG3C 0.4000 2 180.00 ! 2PDO, 2-pyrrolidinone

OG2D CG2R NG2R CG3C 2.5900 2 180.00 ! 2PDO, 2-pyrrolidinone

NG2R CG2R NG2R CG3C 7.7000 2 180.00 ! 7.7 2IMP, 2-imidazoline.H+

NG2R CG2R CG3C HGA1 0.08000 3 0.00 ! 2PDO, 2-pyrrolidinone

NG2R CG2R CG3C CT2 1.0500 3 0.00 ! 2PDO, 2-pyrrolidinone

HGP1 NG2R CG2R CG3C 1.2700 2 180.00 ! 2PDO, 2-pyrrolidinone

CG2R CG3C CT2 HA 0.2000 3 0.00 ! par22, X CT1 CT2 X; erh 3/08

CG2R CG3C CT2 CA 1.6000 3 0.00 ! par22, X CT1 CT2 X; erh 3/08

OG2D CG2R CG3C HGA1 0.08000 3 180.00 ! 2PDO, 2-pyrrolidinone

NG2R CG2R NG2R CG2R 7.7000 2 180.00 ! 7.7 2IMP, 2-imidazoline.H+

OG2D CG2R CG3C CT2 0.0800 3 0.00 ! 2PDO, 2-pyrrolidinone

HGA1 CG3C CT2 HA 0.2000 3 0.00 ! par22, X CT1 CT2 X; erh 3/08

HGA1 CG3C CT2 CA 0.2000 3 0.00 ! par22, X CT1 CT2 X; erh 3/08

CG3C CT2 CA CA 0.2300 3 0.00 ! ALLOW ARO ALI

HA CT2 CA CA 0.2300 2 180.00 ! ALLOW ARO ALI

!!! Improper torsion

CG2R NG2R NG2R OG2D 90.000 0 0.00

CG2R CG3C NG2R OG2D 90.00 0 0.00

CA CT2 CA CA 30.0 0 0.00

References:

Anisimov VM, Lamoureux G, Vorobyov IV, Huang N, Roux B et al. (2005) Determination of electrostatic parameters for a polarizable force field based on the classical Drude oscillator. Journal of Chemical Theory and Computation 1(1): 153-168.

Vanommeslaeghe K, Hatcher E, Acharya C, Kundu S, Zhong S et al. (2010) CHARMM General Force Field: A Force Field for Drug-Like Molecules Compatible with the CHARMM All-Atom Additive Biological Force Fields. Journal of Computational Chemistry 31(4): 671-690.
