# Supplementary figures and images for: The Molecular Mechanism of Ion-Dependent Gating in Secondary Transporters
Source: PLoS Comput Biol. 2013 Oct 24;9(10):e1003296. doi: 10.1371/journal.pcbi.1003296 (PMC3812048; doi:10.1371/journal.pcbi.1003296)

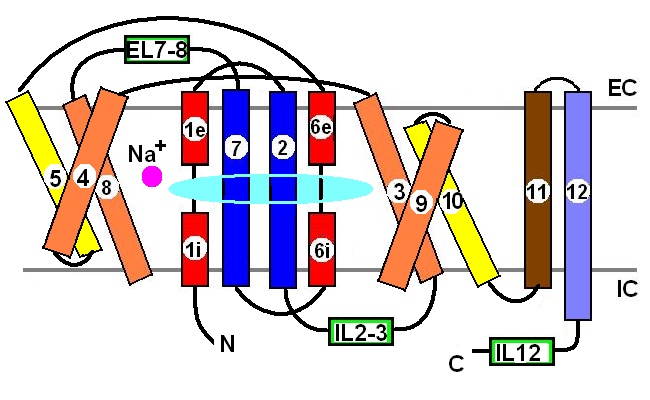

Supplement: Figure S1 — Topology of Mhp1. The “bundle” helices (TM's 1,2,6,7) are shown in red and blue. The “hash motif” (TM's 3,4,8,9) helices are shown in orange. They form a hash “#” sign and thus the name. Na2 ion is shown in magenta. The site is formed by TM1 and TM8, at the interface of the “bundle” and the “hash motif”. The substrate binding site is illustrated as blue oval [11], [31]. (TIF) [file pcbi.1003296.s002.tif]

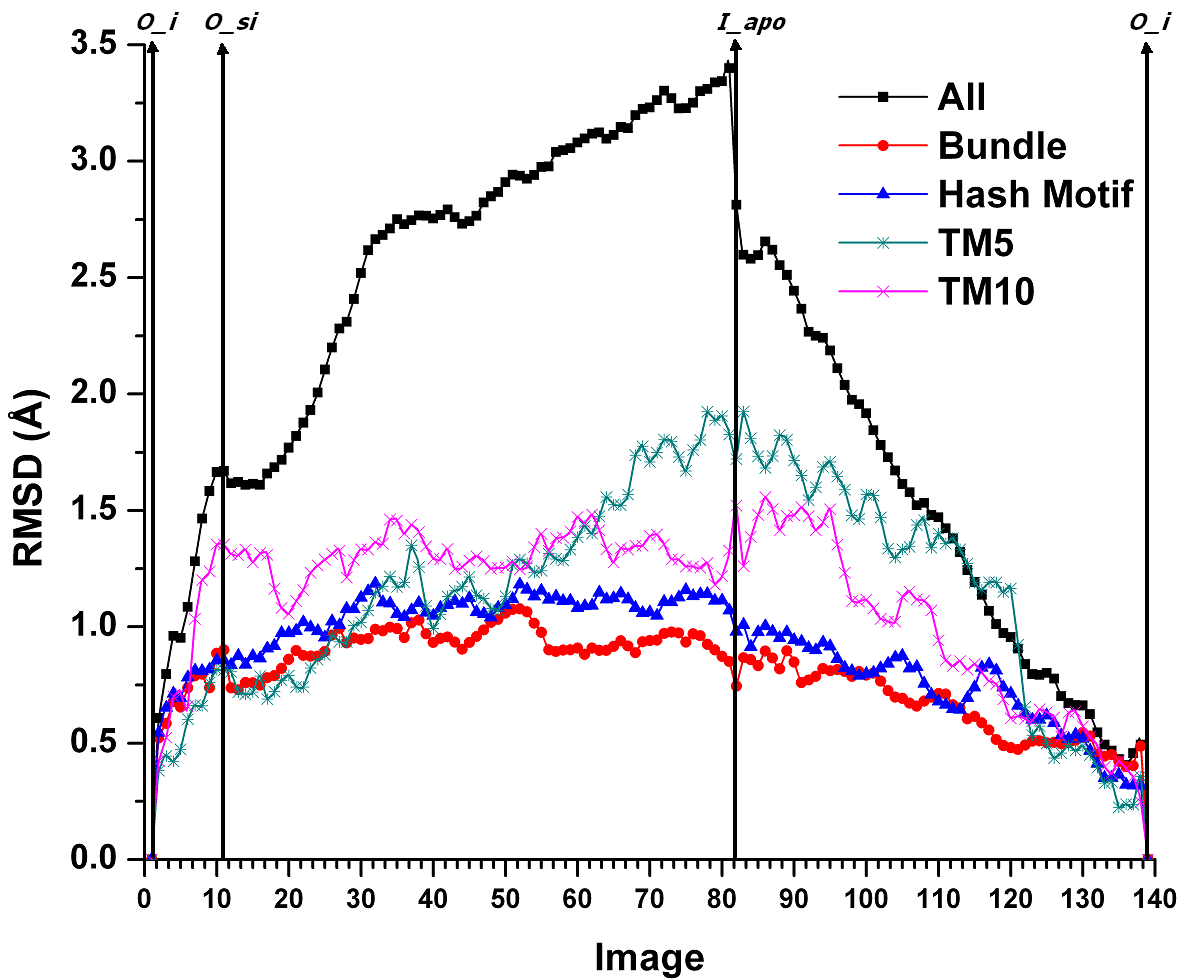

Supplement: Figure S2 — RMSD changes along the transition path from string method with swarm of trajectories. The RMSD values for the backbone heavy atoms of the residues between each image on the path and the reference image (O_i) are computed after a RMSD best-fit of the residues in selection. The RMSD values for residues ARG10 to GLY470 (All), the bundle (TM's 1,2,6,7), the hash motif (TM's 3,4,8,9), TM5, and TM10 are plotted in black squares, red circles, blue triangles, green stars, and purple crosses respectively. (TIF) [file pcbi.1003296.s003.tif]

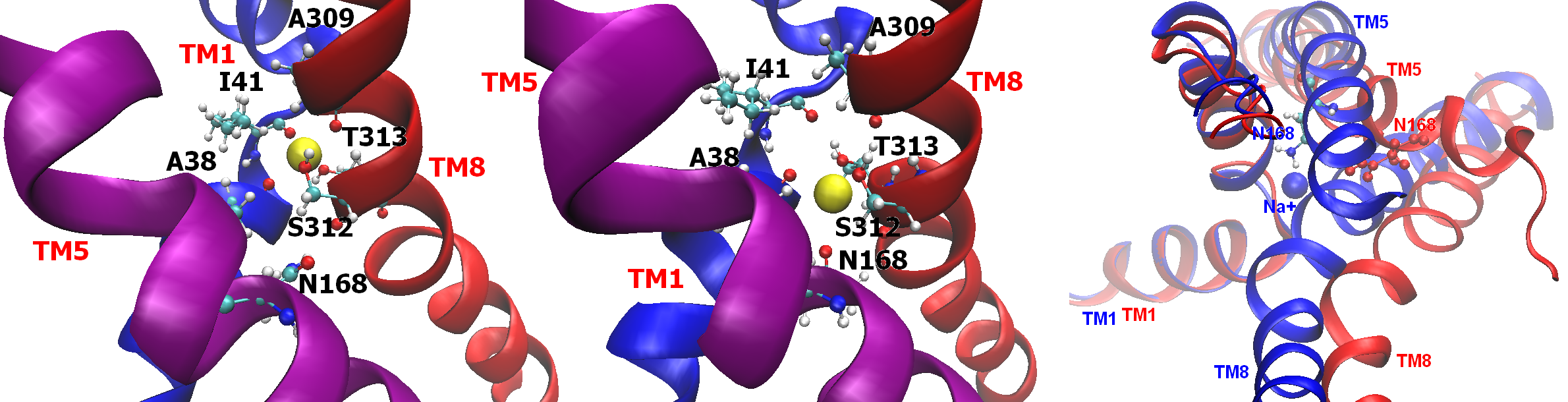

Supplement: Figure S3 — The Na2 (left panel) and the proposed Na2' (centre panel) sites and its effect on inward-facing gating. Structures are taken from umbrella sampling simulations. The left panel represents a typical conformation (Na+ in Na2) at the global minimum in Fig. 3A while the centre panel represents a typical conformation at the nearby minimum when the Na+ moves down by ∼2 Å, coordinated by Asn168 of TM5 (Na+ in Na2'). In the right panel, the effect of Na bound and unbound on the intracellular gating is shown by comparing the O_si (blue) and I_apo (red) structure. In the I_apo structure, TM8 and TM5 move relatively away from TM1 to open up the ion release pathway to the IC bulk. (TIF) [file pcbi.1003296.s004.tif]

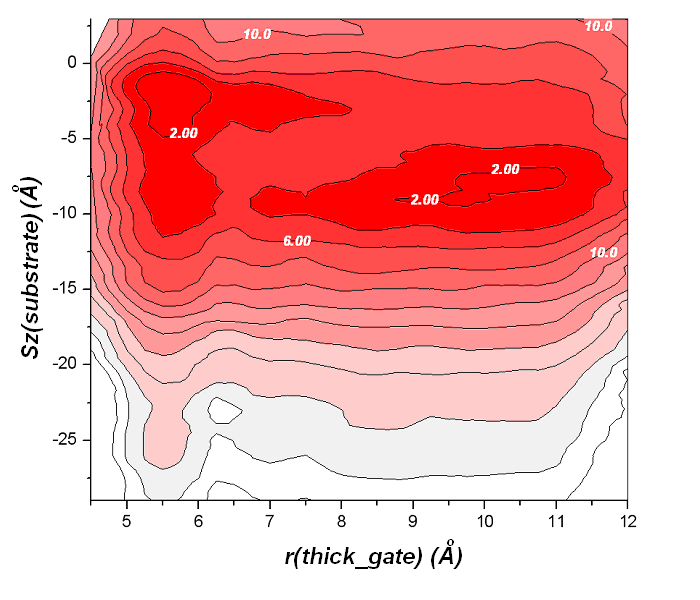

Supplement: Figure S4 — Releasing of the substrate from occluded state (O_si). Conformational distribution of the Mhp1 thick gate highlighting the transition between outward-facing occluded (O_si) and inward-facing open (I_apo). The figure depicts the potential of mean force landscapes governing the open/closure of the thick gate (r(thick_gate)) as a function of the release of the substrate (Sz(substrate)) calculated from all-atom umbrella sampling MD simulations with explicit membrane and solvent. Each contour line corresponds to 2 kcal/mol. PMF value of selected contour lines are marked with white numbers. (TIF) [file pcbi.1003296.s005.tif]

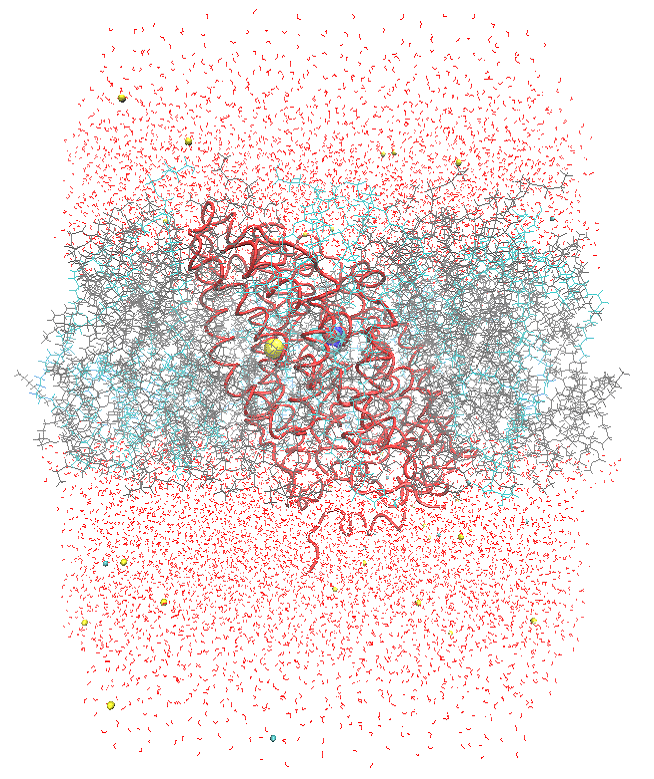

Supplement: Figure S5 — Simulation cell for the outward-facing occluded Mhp1 with both the Na+ (shown as a big yellow ball) and substrate bound (shown as a collection of cyan, white, red, and blue balls). The protein is shown in red tubes. Lipids POPE (grey) and POPG (cyan) are shown in lines. Water molecules, Na+, and Cl− counter ions are shown in red lines, small yellow balls, and small cyan balls respectively. All molecular figures, including this one here, are generated with VMD [56]. (TIF) [file pcbi.1003296.s006.tif]

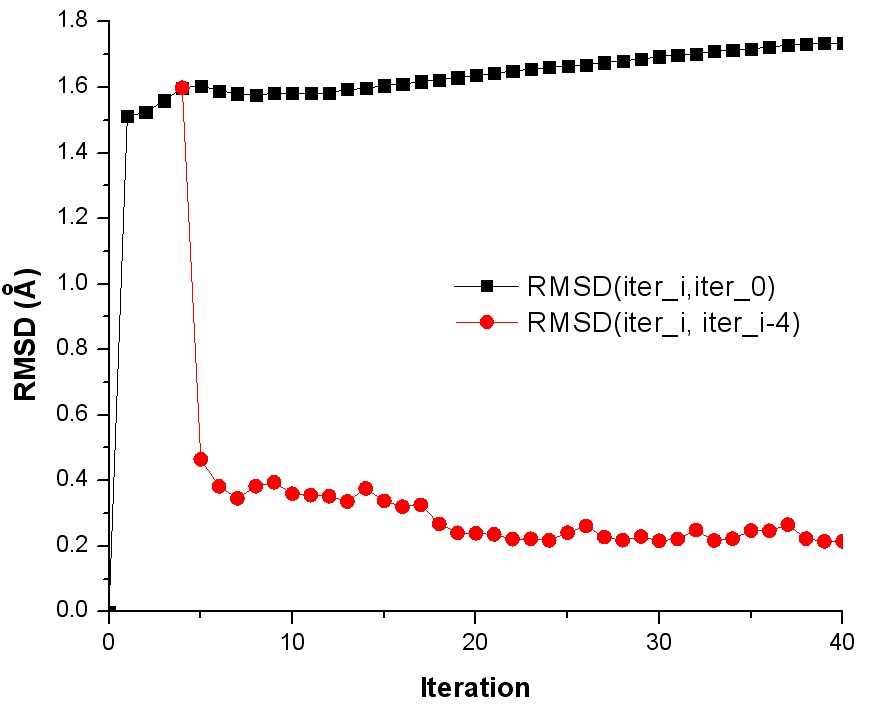

Supplement: Figure S6 — Convergence of the swarm-of-trajectories string method transition path. Average RMSD between the images in the current iteration (iter_i) and their corresponding images in the initial path (iteration 0) as a function of iteration number is plotted in black squares for the transition of O_si to I_apo. Average RMSD between the images in the current iteration (iter_i) and their corresponding images 4 iterations before (iteration i-4) as a function of iteration number is plotted in black squares. Both lines approaches plateaus and convergence is assumed [54]. (TIF) [file pcbi.1003296.s007.tif]

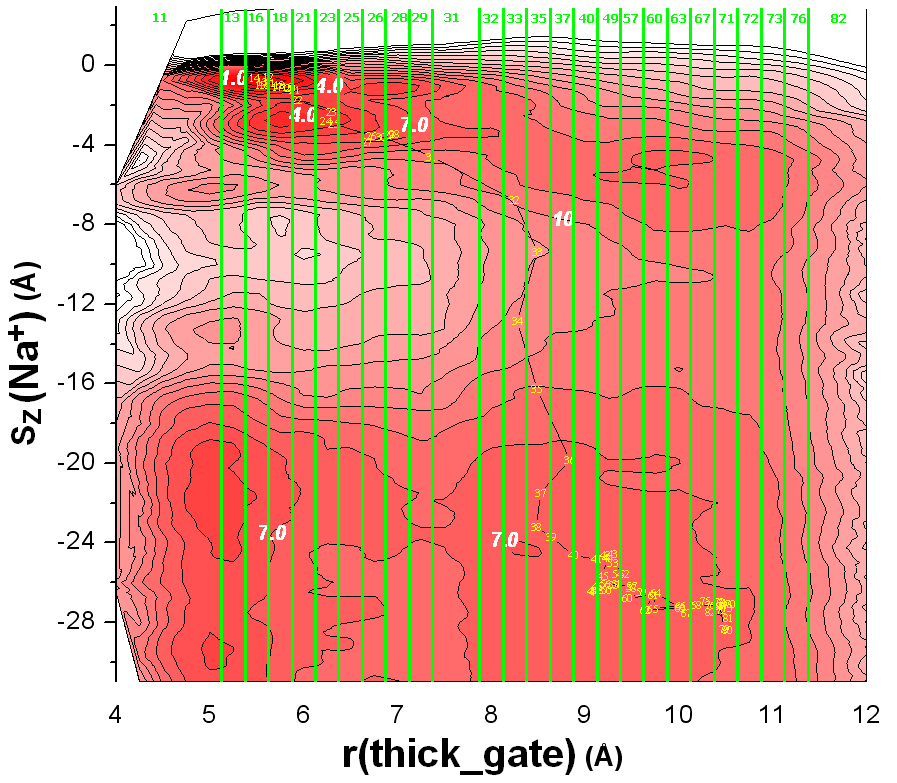

Supplement: Figure S7 — Mapping of the transition path obtained from string method to the 2D PMF about Sz(Na+) and r(thick_gate_distance) for the transition of O_si to I_apo and the illustration for selection of starting conformation for umbrella sampling simulations. The background 2D-PMF shows the free energy landscapes governing the open/closure of the thick gate (r(thick_gate)) as a function of the binding of the Na+ (Sz(Na+)) (Figure 3A). Each contour line corresponds to 1 kcal/mol. PMF value of selected contour lines is marked with white numbers. The landscapes vary from red to white with darker colors indicating more favorable conformation. The transition path from string method is projected to the 2D map with each intermediate structure shown with an orange number on the 2D map (number 11–82 represent the initial Image 11 to final Image 82). The orange numbers above the 2D map indicate the selection of the conformational structure for the umbrella sampling for sampling windows sit between the two green vertical lines. For example, for the umbrella sampling windows between r(thick_gate) 3.875 Å and 5.125 Å, Image 11 is used as the starting conformation. (TIF) [file pcbi.1003296.s008.tif]

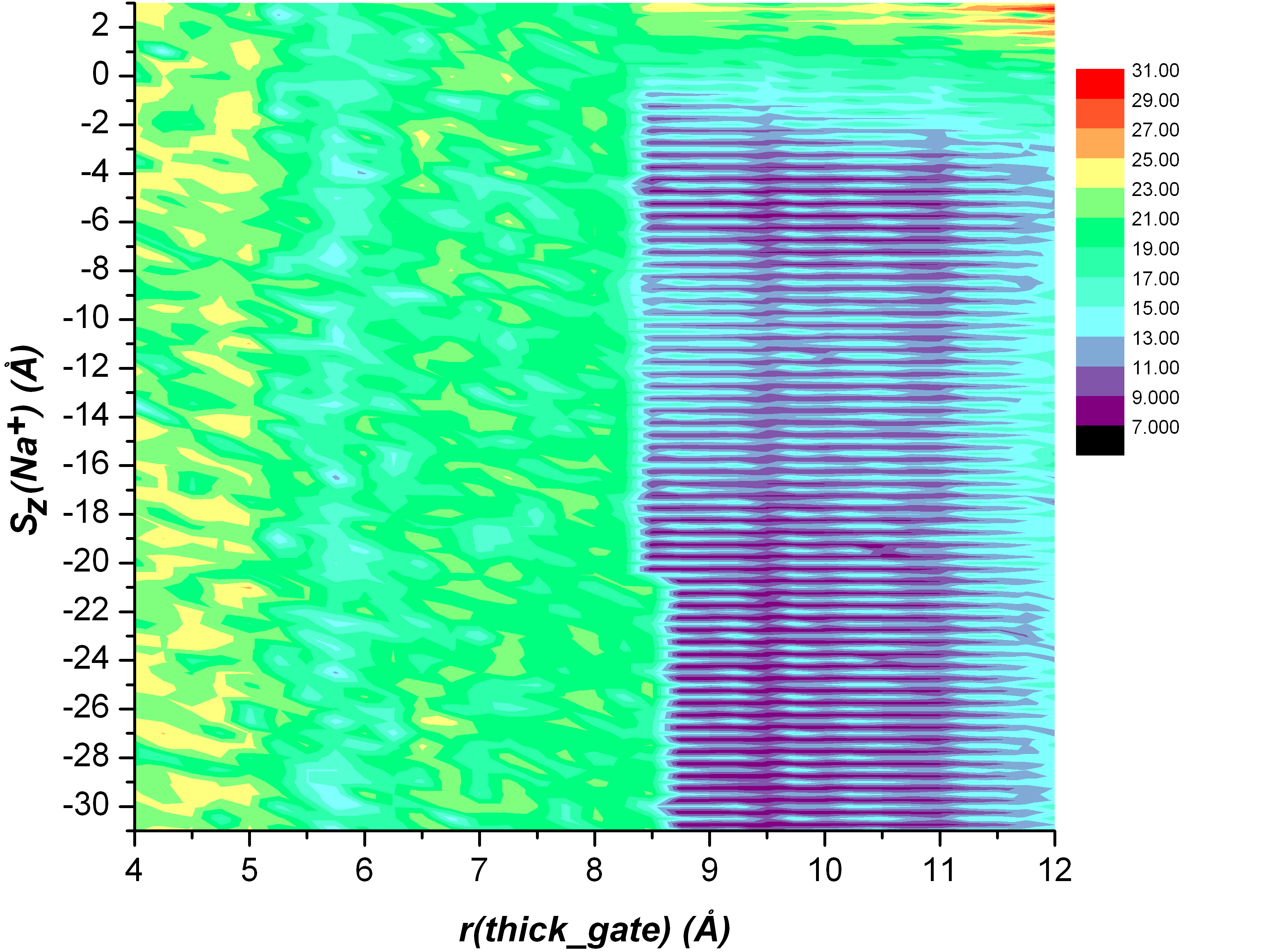

Supplement: Figure S8 — The distribution of minimal distances between Na+ ions present in the system and any atom of the residues (A38, I41, A309, S312, T313) forming the sodium binding site for each (2-dimensional) window computed from the trajectories of the umbrella sampling simulations. The minimal distance distributions are from 7 Å to 32 Å for all the windows of umbrella sampling simulations leading to the 2D-PMF in Figure 3A. Na+ are not contaminating apo-state simulations. (TIF) [file pcbi.1003296.s009.tif]
