# Supplementary material for: The Molecular Mechanism of Ion-Dependent Gating in Secondary Transporters
Source: PLoS Comput Biol. 2013 Oct 24;9(10):e1003296. doi: 10.1371/journal.pcbi.1003296 (PMC3812048; doi:10.1371/journal.pcbi.1003296)
Supplement: Table S1 — Definition of order parameters used in this paper. “COM” stands for Center Of Mass. z() represents the z component of the Cartesian coordinates. (DOC) [file pcbi.1003296.s010.doc]

**Supplemental Table S1:** Name, description, and definition of order parameters. “COM” stands for Center Of Mass. z() represents the z component of the Cartesian coordinates

| Name | Description | Definition |
| --- | --- | --- |
| r(thick_gate) | Na^+^ site distance | COM(Ala38, Ile41) COM(Ala309, Ser312, Thr313) |
| r(EC_thin_gate) | Extracellular thin gate distance | COM(Ile47, Ala48, Ala49) COM(Ala360, Thr361, Phe362) |
| Sz(substrate) | Substrate relative z position to its site | z(COM(Substrate))-z(COM(Trp117, Gln121, Asn318)) |
| Sz(Na^+^) | Na^+^ relative z position to its site, only the backbone oxygen’s are included as a reference because they are relatively less mobile. | z(Na^+^)-z(COM(Ala38 O, Ile41 O, Ala309 O)) |
